# STING Operation at the ER/Golgi Interface

**DOI:** 10.3389/fimmu.2021.646304

**Published:** 2021-05-03

**Authors:** Tomohiko Taguchi, Kojiro Mukai, Eiko Takaya, Ruri Shindo

**Affiliations:** ^1^Laboratory of Organelle Pathophysiology, Department of Integrative Life Sciences, Graduate School of Life Sciences, Tohoku University, Sendai, Japan; ^2^AMED-PRIME, Japan Agency for Medical Research and Development, Tokyo, Japan

**Keywords:** STING, trans-Golgi network, palmitoylation, retrograde membrane traffic, COPA syndrome, SAVI, innate immunity, STING regulation by membrane traffic

## Abstract

DNA is present in the nucleus and mitochondria of eukaryotic cells. There are, however, certain instances in which DNA emerges in the cytosol. The two major sources of cytosolic DNA are self DNA that is leaked out from the nucleus or mitochondria, and non-self DNA from DNA viruses. The cytosolic DNA triggers the host immune response. Recent studies have identified two key molecules, cyclic GMP-AMP (cGAMP) synthase (cGAS) and stimulator of interferon genes (STING) in this immune response. STING is an endoplasmic reticulum (ER) protein. After STING binding to cGAMP, STING exits the ER and translocates to the Golgi, where STING triggers the type I interferon- and proinflammatory responses through the activation of interferon regulatory factor 3 (IRF3) and nuclear factor-kappa B (NF-κB). STING also activates other cellular responses including cell senescence, autophagy, and cell death. In this review, we focus on emerging issues regarding the regulation of STING by membrane traffic, with a particular focus on the retrograde membrane traffic from the Golgi to the ER. The retrograde membrane traffic is recently shown by us and others to be critical for silencing the STING signaling pathway and the defect in this traffic underlies the pathogenesis of the COPA syndrome, a monogenic autoinflammatory disease caused by missense mutations of coatomer protein complex subunit α (COP-α).

## Introduction

The innate immune response is essential for efficient and rapid host defense against invading pathogens. Invading pathogens are sensed by pattern recognition receptors (PRRs) in the host cell (1, 2). PRRs include Toll-like receptors (3), RIG-I-like receptors (4), and nucleotide-binding domain and leucine-rich repeat-containing receptors (5), C-type lectin receptors (CLRs) (6). They bind microbial molecules such as CpG DNA, viral RNAs, and lipopolysaccharides. Activated PRRs initiate a series of intercellular signaling events, leading to the production of type I interferons, proinflammatory cytokines, and antiviral proteins that all coordinate to eliminate pathogens and infected cells.

An ER-associated molecule referred to as STING (7), also known as MITA (8), ERIS (9), MPYS (10), or TMEM173, has been shown to contribute to a sensing pathway that is critical for detecting cytosolic DNA or cyclic dinucleotides (CDNs) (11) including cGAMP (12). CDN-bound STING translocates from the ER to the Golgi, where STING recruits TBK1 (13, 14), which then phosphorylates IRF3. Phosphorylated IRF3 dimerizes and translocates to the nucleus to induce transcription of genes that encode type I interferons such as interferon-β (IFNβ). STING also induces proinflammatory response *via* NF-κB by the activation of TBK1 and IKKϵ (15, 16). Inborn errors of innate immunity that is linked to dysregulated activation of cGAS/STING/TBK1/IRF3 have been described in multiple autoinflammatory or neurodegenerative diseases [see the review by (17)], such as Aicardi-Gourieres syndrome, systemic lupus erythematosus, Parkinson disease, and amyotrophic lateral sclerosis. These findings underscore the critical roles of the STING pathway in human pathophysiology.

Several PRRs including TLRs, CLRs, and STING are transmembrane proteins. They localize at various organelles, such as the plasma membrane (PM), late endosomes, recycling endosomes (REs), and the ER. Upon binding to their ligands, some PRRs relocate to other organelles by membrane traffic and trigger the innate immunity signaling there. Not surprisingly, impaired membrane traffic of PRRs often makes the host susceptible to infection or prone to autoinflammatory diseases. These findings emphasize the critical role of membrane traffic in innate immunity. In this review, we focus on emerging issues regarding the regulation of STING activity by membrane traffic, with a particular focus on the membrane traffic between the ER and the Golgi. This membrane traffic is recently shown to relate to two autoinflammatory diseases, STING-associated vasculopathy with onset in infancy (SAVI) (18, 19) and the COPA syndrome (20), which is caused by missense mutations of coatomer protein COP-α.

## Exocytic Membrane Traffic of STING From the ER

After the binding of STING to CDNs, STING relocates from the ER to the Golgi (21, 22). Treatment with brefeldin A (BFA) or expression of *Shigell*a effector IpaJ, which inhibits the ER-to-Golgi traffic, dampens the phosphorylation of IRF3 and induction of IFNβ, suggesting that the ER-to-Golgi traffic is required for the STING signaling (13, 21, 23, 24). The ER-to-Golgi traffic is facilitated by the coat protein complex-II (COP-II), a protein complex that is responsible for creating membrane vesicles (COP-II vesicles) that bud from the ER (25). Sar1 (a small GTPase), Sec23/Sec24 (inner coat proteins), and Sec13/Sec31 (outer coat proteins) are five cytosolic components of the COP-II complex. Knockdown of Sar1A and Sar1B, two mammalian Sar1 paralogs, inhibited the STING translocation from the ER to the Golgi and the phosphorylation of IRF3, indicating that the exit of STING from the ER with COP-II vesicles is required for the STING signaling (14). The critical role of COP-II-mediated transport of STING in the STING signaling was corroborated by the experiments with knockdown of Sec13, Sec23, Sec24, or Sec31 (26–28).

Several proteins associated with COP-II are also involved in the STING signaling. Yip1 domain family (YIPF) proteins are multi-spanning membrane proteins (29). Yeast Yip1p regulates COP-II vesicle biogenesis (30). YIPF5 (a mammalian ortholog of yeast Yip1p) is responsible for cytosolic DNA-induced translocation of STING from the ER and the induction of IFNβ (27). The transmembrane emp24 domain (TMED) family are single-spanning transmembrane proteins and have emerged as critical regulators of the early (ER-to-Golgi) and late (Golgi-to-plasma membrane) exocytic pathways (31). Yeast Emp24p and Erv25p belong to TMED family and both proteins recruit specific cargo molecules into COP-II vesicles (32, 33). TMED2 (a mammalian ortholog of yeast Emp24p) is required for herpes simplex virus (HSV)-induced translocation of STING from the ER and the STING signaling (26). TMED10 (a mammalian ortholog of yeast Erv25p) also participates in the STING signaling (26).

The rhomboid family are evolutionary conserved intramembrane proteases. Mammalian iRhom1 and iRhom2, do not possess the protease activity and are hence dubbed “inactive” rhomboid family members (34). iRhom2 localizes at the ER and is essential for the translocation of a disintegrin and metalloproteinase 17 (ADAM17) from the ER to the Golgi (35). iRhom2 is required for HSV-induced translocation of STING from the ER and the STING signaling (36). Recently, CxORF56, or STING ER exit protein (STEEP), is shown to facilitate the STING trafficking from the ER. After cGAMP stimulation, STEEP stimulates phosphatidylinositol-3-phosphate [PtdIns(3)P] synthesis in the ER membrane and induces membrane curvature at the ER (37). Whether PI(3)P-dependent membrane curvature formation promotes COP-II vesicle biogenesis remains to be elucidated.

**Figure 1:**
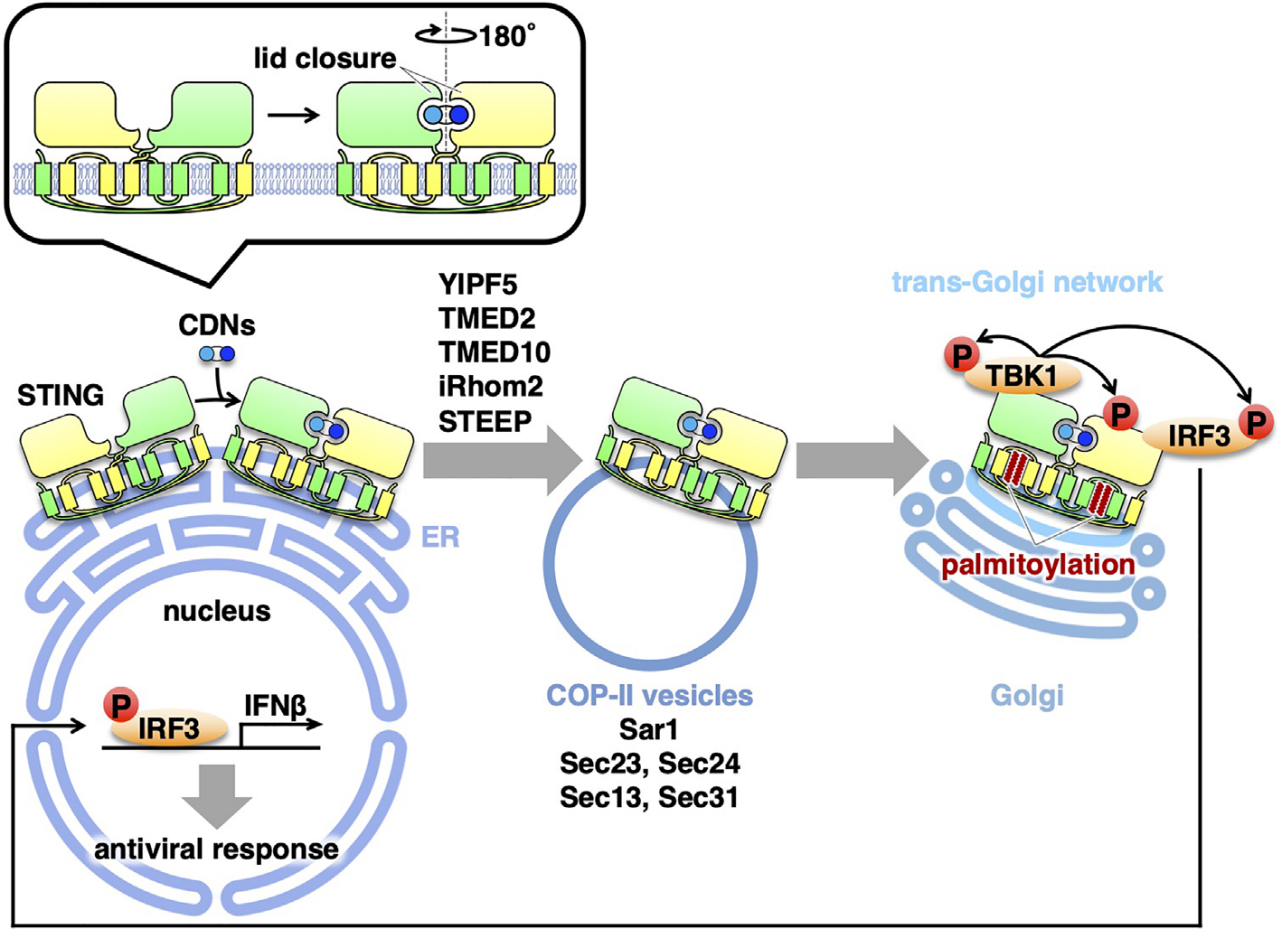
Exocytic membrane traffic of STING from the ER. STING localizes at the ER at the steady-state. Upon binding to CDNs, STING translocates from the ER to the Golgi with COP-II vesicles. Several proteins (YIPF5, TMED2, TMED10, iRhom2, and STEEP) associated with the COP-II components are required for the translocation of STING. After reaching the Golgi, STING undergoes palmitoylation and activate TBK1 at the trans-Golgi network.

How does the binding of cGAMP to STING promote the translocation of STING? One clue is provided by the cryo-electron microscopy structure of cGAMP-bound STING (38). The cGAMP binding causes a 180° rotation of the ligand-binding domain of STING relative to its transmembrane domain (the inset in **Figure 1**). This rotation affects a conformation of a loop on the side of the ligand-binding-domain dimer, leading to the formation of the STING oligomers. Another study suggests that the oligomerization of STING requires Cys148-mediated disulfide bridges (39). These conformational changes of STING will expose and/or generate the binding interface(s) with the COP-II components and/or the proteins that regulate COP-II vesicle biogenesis.

All of the molecules described above are positive regulators of the STING trafficking from the ER with COP-II vesicles. Recently, the Ca^2+^ sensor stromal interaction molecule 1 (STIM1), an ER-resident protein, is shown to be a suppressor of the STING signaling (40). STIM1 deficiency results in a low-level induction of IFNβ and a partial STING translocation to the perinuclear compartment without cGAMP stimulation. The induction of IFNβ is abolished by concurrent knockout of STING. Importantly, STIM1 binds STING under unstimulated conditions, and its interaction with STING is reduced upon cGAMP binding. Therefore, STIM1 serves as a tether to retain STING at the ER under steady/unstimulated conditions. Thus, there appears a “tug-of-war” of STING at the ER membrane between the COP-II components and the tethering molecule(s), and their power balance may be critical for STING dynamics. The conformational change of STING upon cGAMP binding should influence the power balance, favoring the STING exit from the ER with the COP-II components.

## STING Activation at the Golgi

As mentioned above, the trafficking of STING from the ER is critical to activate the downstream signaling cascade. Recent evidence suggests that palmitoylation of STING at the Golgi is essential for activation of STING (13). Treatment of cells with palmitoylation inhibitor 2-bromopalmitate (2-BP) inhibits type I interferon response without affecting the trafficking of STING from the ER. Mutation of two membrane-proximal Cys residues (C88/91) suppresses palmitoylation, and this STING variant is incapable of inducing STING-dependent signaling. The significance of C88/91 in STING signaling is corroborated by the recent findings of identification of STING inhibitors that target C88/91 of STING (41–43). Of note, the scrutinization of the Golgi with immunofluorescence microscopy suggests that the activation of TBK1 occurs exclusively at the trans-Golgi network (TGN) (13), a Golgi domain that is responsible for the sorting of exocytic cargo molecules for delivery to the PM and endosomes.

Protein palmitoylation has been implicated in the assembly of proteins (44) into lipid rafts, specific lipid microdomains that contain cholesterol and sphingomyelin (SM). At the TGN, cholesterol and SM generated by SM synthase 1 (the Golgi-localized SM synthase) are suggested to form lipid rafts at the TGN (45). C6-ceramide treatment interferes the integrity of lipid rafts at the Golgi by producing short-chain SM (45). C6-ceramide inhibits the STING signaling without affecting the relocation of STING from the ER or palmitoylation of STING (13). Thus, we hypothesize that STING palmitoylation allows STING to cluster in the lipid rafts at the TGN, facilitating the recruitment of TBK1 and IRF3 onto STING. Given that STING can cluster upon cGAMP binding (38), the clustering nature of STING at the TGN may be qualitatively distinct from that of cGAMP-bound STING at the ER, perhaps in the number and/or the spatial arrangement of STING in the cluster.

An autoinflammatory syndrome termed STING-associated vasculopathy with onset in infancy (SAVI) is caused by gain-of-function mutations in STING (18, 19, 46, 47). The SAVI mutations (V147L, N154S, V155M, C206Y, R281Q, and R284G) cause constitutive activation of STING without cGAMP stimulation. These SAVI variants do not stably localize at the ER, instead localize at the perinuclear compartments (13, 14). The suppression of ER-to-Golgi traffic with BFA abolishes TBK1 binding to the SAVI variants (14). The interferon response elicited by these SAVI variants is inhibited by 2-BP or an introduction of Cys88/91Ser mutation (13). These results suggest that the constitutively active SAVI variants even require the translocation from the ER and their palmitoylation at the Golgi for their activity. The experimental 3D modeling of the C-terminal cytosolic domain of STING predicts that the one of the SAVI mutations, V155M, increases the stability of the STING dimer (18). The structure of the SAVI variants may mimic that of cGAMP-bound STING, therefore, the SAVI mutations may skew STING affinity to the COP-II components (37), allowing the SAVI variants to exit the ER without cGAMP binding.

Several studies have suggested that the ER-Golgi intermediate compartment (ERGIC) is the site of STING activation. ERGIC is a complex membrane system that resides between the ER and the Golgi. Conventionally, ERGIC is regarded as ERGIC-53 (p58)-positive compartment. However, ERGIC-53 circulates between the ER/ERGIC/the Golgi, the care has to be taken in interpreting co-localization data of STING and ERGIC-53. As mentioned above, pTBK1, the active form of TBK1, localizes exclusively at the TGN, not at the rest of the Golgi domains (13), arguing against that the ERGIC is the site of STING activation. The role of ERGIC in the STING activation will be firmly demonstrated when one develops the way to inhibit the “ERGIC-to-Golgi” membrane trafficking.

## Retrograde Membrane Traffic of STING From the Golgi

The COPA syndrome is a recently discovered monogenic autoinflammatory/autoimmune disorder characterized by interstitial lung disease and high expression of type I interferon-stimulated genes (20, 48). The disease is caused by heterozygous mutations of the COPA gene, encoding COP-α of the COP-I complex. The COP-I complex mediates the retrograde membrane transport from the Golgi to the endoplasmic reticulum (ER) *via* COP-I vesicles (49, 50). All of the mutations lie in the N-terminal WD40 domain of COP-α, which is implicated in the recognition of cargo proteins (51). How the impaired retrograde transport causes COPA syndrome remained largely unknown.

Recently, four groups provide the evidence that COPA syndrome is caused by constitutive activation of STING, being accompanied with a loss of the ER localization of STING (52, 53). With the disease-causative COP-α variants, STING is not able to be retrieved back to the ER from the Golgi because of the impaired COP-I transport. The forced Golgi localization of STING leads to the activation of STING at the TGN (54). COP-α binds *C*-terminal di-lysine motifs (KKXX and KXKXX) of its cargo proteins (49, 55, 56). Since STING does not have these motifs, we reason the presence of adapter protein(s) that links STING and α-COP. By mass spectrometric analysis, we identify 18 proteins with these motifs. Knockdown of Surf4, not that of the other 17 proteins, relocates STING from the ER to the Golgi and results in the emergence of p-TBK1 (54). STING/Surf4/COP-α complex is disrupted in the presence of the disease-causative COP-α variant. These results suggest that Surf4/α-COP axis is essential to maintain the steady-state localization of STING to the ER. Intriguingly, the binding of the SAVI variants to Surf4 is reduced (54). The reduced binding may partly explain the aberrant localization of the SAVI variants to the Golgi.

Several neurodegenerative diseases are linked to COP-I dysfunction. Mutations in Scy1-like 1 (Scyl1) in mice cause motor neuron degeneration and cerebellar atrophy (57, 58). Scyl1 binds to COP-β subunit of the COP-I complex and knockdown of Scyl1 disrupts COPI-mediated retrograde traffic from the Golgi to the ER (59). Golgi brefeldin A-resistant guanine nucleotide exchange factor 1 (GBF1) is a guanine-nucleotide exchange factor for ADP-ribosylation factor family of small GTPases (60). GBF1 is involved in the formation of COP-I vesicles, maintenance, and function of the Golgi. The pathogenic variants of GBF-1 are recently found in individuals affected by distal hereditary motor neuropathies (HMNs) and axonal Charcot-Marie-Tooth neuropathy (CMT2) (61). It is tempting to speculate that STING is constitutively activated in these neurodegenerative diseases, as in the COPA syndrome, contributing in part to their pathogenesis.

## Concluding Remarks

**Figure 2:**
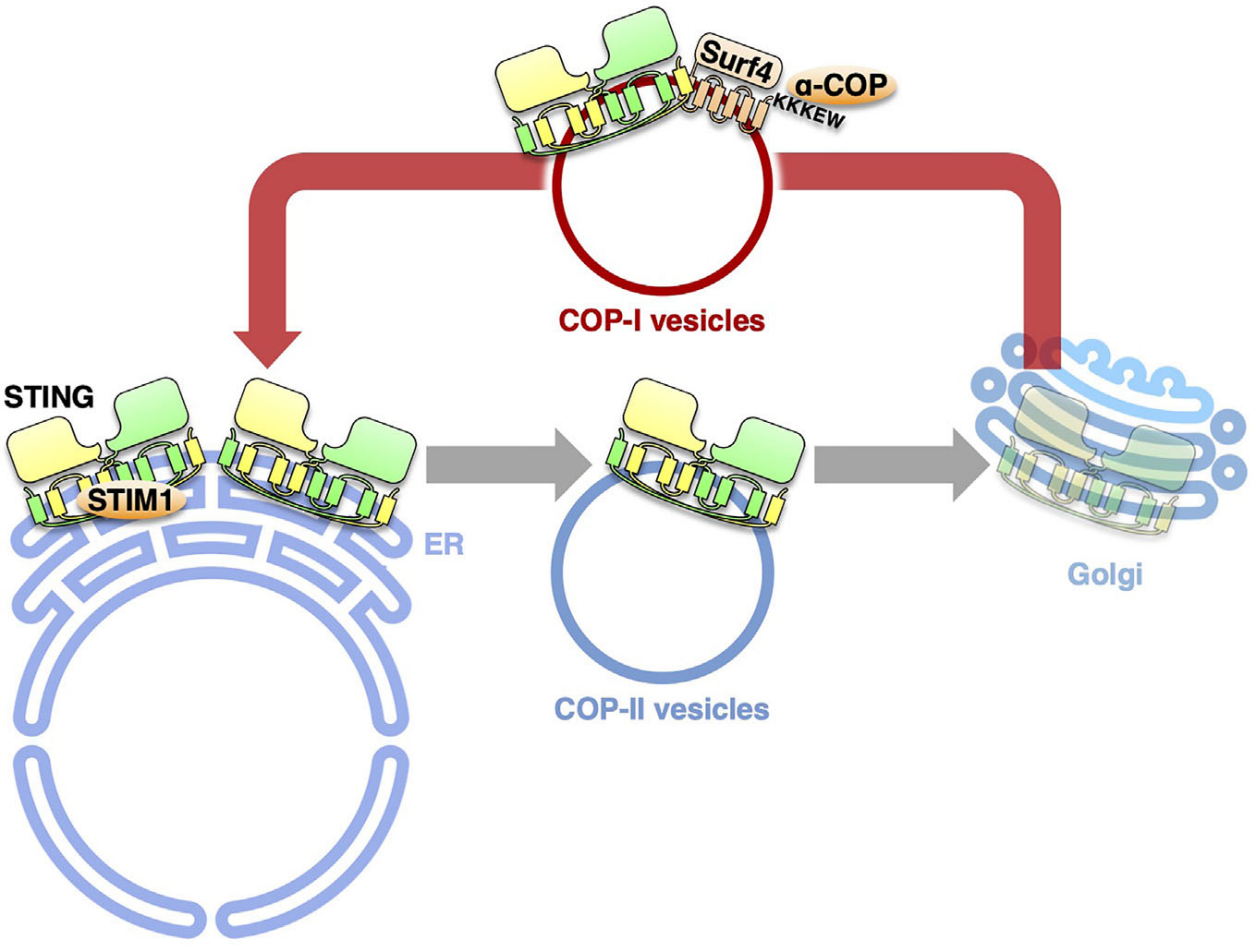
The retrograde membrane traffic retrieves STING from the Golgi to the ER. The retrograde membrane traffic from the Golgi to the ER, which is mediated by COP-I vesicles, suppresses STING activation, by preventing STING from reaching the TGN. Surf4 functions as an adaptor to link STING and α-COP. STIM1 serves as a tether to retain STING at the ER.

During the last several years, substantial progress has been achieved in the molecular mechanism underlying the cGAS/STING pathway, one of the critical innate immune signaling pathways. The activity of STING is now revealed to be strictly regulated by membrane trafficking. The exocytic membrane traffic from the ER, which is mediated by COP-II vesicles, promotes STING activation. In contrast, the retrograde membrane traffic from the Golgi to the ER, which is mediated by COP-I vesicles, suppresses STING activation, by preventing STING from reaching the TGN. Thus, recent studies on the COPA syndrome demonstrate another “tug-of-war” of STING between the ER and the Golgi, which involves the COP-II- and COP-I-mediated membrane transports (**Figure 2**).

Despite these advances, critical questions remain unanswered, such as the nature of palmitoylated STING, the nature of STING oligomers, the molecular mechanism underlying TBK1 recruitment to STING, and the regulators of post-Golgi membrane trafficking of STING to lysosomes. These studies are anticipated to make broad conceptual contributions to cell biology (membrane traffic and signaling), biochemistry (protein lipidation), and innate immunity (autoinflammatory diseases).

## Author Contributions

TT conceptualized the layout of the topics and wrote the review. KM gathered the information over the review’s topics and prepared the figures. ET and RS gathered the information regarding the Golgi-associated diseases. All authors contributed to the article and approved the submitted version.

## Funding

This work was supported by JSPS KAKENHI Grant Numbers JP19H00974 (TT), JP20H05307 (KM), JP20H03202 (KM), and AMED-PRIME (17939604) (TT).

## Conflict of Interest

The authors declare that the research was conducted in the absence of any commercial or financial relationships that could be construed as a potential conflict of interest.
